# Plasma and Cerebrospinal Proteomes From Children With Cerebral Malaria Differ From Those of Children With Other Encephalopathies

**DOI:** 10.1093/infdis/jit334

**Published:** 2013-07-25

**Authors:** Evelyn N. Gitau, Gilbert O. Kokwaro, Henry Karanja, Charles R. J. C. Newton, Stephen A. Ward

**Affiliations:** 1 Centre for Geographic Medicine–Coast, KEMRI-Wellcome Trust Research Programme, Kilifi; 2 African Centre for Clinical Trials; 3 Consortium for National Health Research; 4 Department of Pharmaceutics and Pharmacy Practice, School of Pharmacy, University of Nairobi, Kenyatta National Hospital, Nairobi, Kenya; 5 Liverpool School of Tropical Medicine; 6 Department of Psychiatry, University of Oxford, United Kingdom

**Keywords:** proteomics, *P. falciparum*, cerebral malaria, spectrin, platelet activation

## Abstract

Clinical signs and symptoms of cerebral malaria in children are nonspecific and are seen in other common encephalopathies in malaria-endemic areas. This makes accurate diagnosis difficult in resource-poor settings. Novel malaria-specific diagnostic and prognostic methods are needed. We have used 2 proteomic strategies to identify differentially expressed proteins in plasma and cerebrospinal fluid from children with a diagnosis of cerebral malaria, compared with those with a diagnosis of malaria-slide-negative acute bacterial meningitis and other nonspecific encephalopathies. Here we report the presence of differentially expressed proteins in cerebral malaria in both plasma and cerebrospinal fluid that could be used to better understand pathogenesis and help develop more-specific diagnostic methods. In particular, we report the expression of 2 spectrin proteins that have known *Plasmodium falciparum*–binding partners involved in the stability of the infected red blood cell, suppressing further invasion and possibly enhancing the red blood cell's ability to sequester in microvasculature.

Identifying characteristics that distinguish different encephalopathies occurring in children in malaria-endemic regions can be difficult, especially in resource-poor settings. *Plasmodium falciparum* is often assumed to be the main cause, but there are many other causes of encephalopathy, including bacterial meningitis or viral meningitis [1]. Frequently, no evidence of an infectious agent is found [2], and between 2007 and 2011, 51% of comatose children admitted to Kilifi District Hospital on the Kenyan coast had coma with no cause identified. In addition, an earlier study of Kenyan children with acute encephalopathy found that a significant proportion who fulfilled the World Health Organization definition of cerebral malaria had viruses detected in the cerebrospinal fluid (CSF) [3]. Whereas bacterial meningitis can be excluded by the examination and culture of CSF [4], exclusion of other encephalopathies remains a significant challenge. This seriously confounds studies on the pathophysiology of cerebral malaria and also delays critical decisions on appropriate clinical management. Therefore, we need better ways of identifying children who have or may develop cerebral malaria to facilitate early clinical decisions on management.

We have previously demonstrated the measurable presence of differentially expressed proteins in plasma from a mouse model of cerebral malaria, compared with noninfected mice [5]. To determine whether differentially expressed proteins could be identified in body fluids from patients with cerebral malaria, we undertook a similar proteomic study, using CSF and plasma from children with cerebral malaria, and compared the protein profiles in these biological matrices with those in samples from children with confirmed acute bacterial meningitis and other nonspecific encephalopathies.

## MATERIALS AND METHODS

### Subjects

The study used archived plasma and CSF samples collected from clinically well-characterized children attending Kilifi District Hospital between 2001 and 2002. The Kenya Medical Research Institute Ethics Review Committee approved all studies. The children came from a geographic region described in detail elsewhere [6]. Children were grouped according to the results of a malaria slide, CSF leukocyte count, and microbiological findings [6]. Cerebral malaria (n = 12) was defined according to World Health Organization criteria: (1) a Blantyre coma score of <3 in the presence of peripheral asexual malarial parasites on the blood film, and (2) negative results of CSF or blood cultures and a CSF leukocyte count of ≤10 cells/μL. In addition, we selected patients with a parasitemia of >2500 parasites/µL, since this cutoff has the most specificity in this area [6]. Acute bacterial meningitis (n = 12) was defined as the absence of asexual malaria parasites in 3 slides of blood specimens obtained over 24 hours; the presence of a CSF leukocyte count of >10 cells/μL or a positive CSF culture result, a positive blood culture result, or detection of bacterial antigen in CSF [1]. Nonspecific encephalopathy (n = 12) was defined as impaired consciousness, no detection of asexual-stage parasites in 3 slides of blood specimens obtained over 24 hours, and no growth on CSF or blood cultures. Matched CSF and plasma samples and complete medical records were available from each patient.

### Sample Preparation

Archived CSF samples that had been stored at −80°C for 5–6 years were thawed at 4°C and desalted using Micorocon YM-3 centrifugal units (Millipore, United States). Archived plasma samples stored at −80°C were also thawed at 4°C. Protein concentrations for the plasma (1:100 v/v dilution with water) and desalted CSF sample were determined using the Bradford assay as previously described [5]. Before storage, CSF samples were centrifuged at 450 ×*g*. All but the bottom 0.5 mL of the supernatant was transferred to new container and stored at −80°C. For plasma, heparinized blood was centrifuged at 450 ×*g* and the plasma was removed, aliquoted, and stored at −80°C.

### 2-Dimensional Gel Electrophoresis

Proteins were separated using 2-dimensional gel electrophoresis and analyzed as previously described [5]. Master gels were prepared by analyzing duplicate gels of samples from 12 individual patients. Spots matched in >75% of the gels were included in the master gel, using PDQuest 2-dimensional software, with semiquantitative analysis performed using Progenesis 200 software. For plasma samples, protein spots of interest were excised from Coomassie-stained gels and digested as previously described [5]. Because of the low protein content in CSF samples, Coomassie-stained gels were not prepared, and spots were cut directly from silver-stained gels and digested using a modified method previously described by Terry et al [7]. Mass spectra of tryptic digests were obtained using a MALDI-ToF mass spectrometer (MS; Shimadzu CFR Plus, Manchester, United Kingdom) as previously described [5]. When definitive protein identification could not be formally made by MALDI-ToF, the tryptic digests of the spots of interest were further separated by reverse-phase–high-performance liquid chromatography performed on an UltiMate 3000 LC system (Dionex, United Kingdom). A total of 1 µL of the concentrated sample was diluted with 4 µL of 2.5% v/v acetonitrile in water containing 0.1% formic acid and injected onto a monolithic capillary column (200-µm internal diameter × 5 cm; Dionex). Peptides were eluted at a flow rate of 1.5 µL/minute, using a solvent gradient of solvent A (2.5% v/v acetonitrile in water with 0.1% formic acid) and solvent B (90% v/v acetonitrile in water with 0.1% formic acid), starting at 5% solvent B, linearly ramped to 40% solvent B over 12 minutes, and then to 90% solvent B for a further 2 minutes. Solvent B was then decreased to 5%, and this was maintained to the end of the run at 27 minutes. Resulting ions were eluted into a LCQ Deca XP Plus ion trap MS (ThermoFinnigan, United States) equipped with a nanospray source connected to a PicoTip column. Further details of the methods used can be found in the Supplementary Materials.

### Two-Dimensional Liquid Chromatography Tandem MS (LC-MS/MS)

The protein separation on gels was subsequently replaced by a 2-dimensional LC-MS/MS–based approach. An equivalent of 100 µg of CSF protein or 200 µg of plasma protein was injected onto a ProSwift RP-1S monolith column (4.6 × 50 mm, Dionex). The high-performance liquid chromatography was performed on an UltiMate 3000 LC system (Dionex UK). Samples were eluted with Solvent A, 2.5% acetonitrile in water with 0.1% trifluoroacetic acid and solvent B, and 90% acetonitrile in water with 0.1% trifluoroacetic acid. The flow rate was maintained at 200 µL/minute, and the separated proteins were eluted into a 96-well plate. Forty-eight fractions per sample were collected. The fractions were then dried down overnight in an oven set at 50°C. A total of 25 µL of 100 mM ammonium bicarbonate was added to the sample, followed by 5 µL of a 20-µg/mL solution of trypsin in 25 mM ammonium bicarbonate. Samples were thoroughly mixed and incubated overnight at 37°C to achieve complete digestion. The resulting digest was subjected to reverse-phase–high-performance liquid chromatography MS as described above.

### *P. falciparum* Histidine-Rich Protein 2 (HRP2) Double-Site Antigen-Capture Enzyme-Linked Immunosorbent Assay (ELISA)

ELISA was used to determine the presence of *p*HRP2 in frozen plasma and CSF samples. Plates were coated with 100 µL/well of 1.0 µg/mL immunoglobulin M monoclonal anti-HRP2 antibody (MPFM-55A, Immunology Consultants Laboratories, Newberg, OR) diluted in phosphate-buffered saline (PBS) and incubated overnight at 4°C. Plates were saturated for 2 hours at room temperature with 200 µL of 3% skimmed milk (Marvel; catalog no. UKFF 005M EC) in PBS. Plates were then washed 3 times in PBS/Tween (Sigma; catalog no. P1379; 500 mL; 0.05%) washing solution. A total of 100 µL of diluted plasma samples (1:64) or 100 µL of CSF samples was added to the plates, which were sealed and incubated at room temperature in a humid chamber for 2 hours and then washed 5 times. A total of 100 µL of secondary antibody conjugated with horseradish peroxidase (MPFG-55P, Immunology Consultants Laboratories; 0.2 μg/mL diluted in 2% bovine serum albumin, 1% Tween 20, and PBS) was added to the wells and incubated for 1 hour at room temperature. After incubation, substrate (Sigmafast OPD; catalog no. P9187-50SET) was added and incubated for 30 minutes at room temperature, and the reaction was stopped by adding 50 µL of 2N sulfuric acid. Plates were read at an optical density of 490 nm. Standards were made by serially diluting plasma samples of known parasitemia, with a high parasitemia of 0.2% and a low of 0.003125% (this gave a 0.1 absorbance value above baseline). A cutoff of 0.025 in plasma and 0.004 in CSF had been determined to separate children with cerebral malaria from all other children with impaired consciousness in a separate group of children.

### Data Management and Statistical Analysis

Differences in clinical characteristics of the 3 disease phenotypes were evaluated using Stata, version 11.2. Medians were calculated, and Kruskal–Wallis *P* values reported.

Spectra obtained from the MALDI-ToF were used to search through the NCBI*nr* database, using the Mascot Peptide Mass Fingerprinting software [8]. Protein scores were considered significant in accordance with cutoff scores recommended by Mascot for *Homo sapiens* 65 *P* < .05 or for *P. falciparum* 55 *P* < .05.

MS/MS spectra were evaluated using the TurboSEQUEST algorithm in BioWorks v 3.1 software provided by ThermoFinnigan and were searched against the human and *P. falciparum* subsets of the NCBInr database and the *P. falciparum* database (PlasmoDB, version 4.4) downloaded from the Sanger Institute. All searches were performed according to search parameters described in the Supplementary Materials. Proteins identified were stored in Stata, version 11.2, and a heat map showing the presence or absence of a protein in a patient was generated using Stata, version 11.2. Proteins were included in the phenotype database if they were identified in samples from at least 6 of the 12 patients in the group.

Functional cataloging of proteins was performed as described elsewhere [5]. Further protein and pathway analysis was undertaken using tools available at the Universal Protein Resource [9].

## RESULTS

### Patient Characteristics

Samples from 36 children were used in this study. Table 1 reports clinical features of the children. As expected, children with acute bacterial meningitis had significantly higher levels of white blood cell counts in the CSF and lower levels of glucose in the CSF. Children with cerebral malaria had significantly fewer platelet counts.

**Table 1.** Clinical Characteristics of Patients

Characteristic	Acute Bacterial Meningitis	Cerebral Malaria	Nonspecific Encephalopathy	*P*^a^
Age, months	32.75 (6.07–86.03)	31.83 (19.98–39.47)	25.55 (13.47–35.50)	.7730
Parasite density, iRBCs ×10^3^/μL	0 (0–0)	566 000 (22 434–1 289 500)	0 (0–56.5)	.0001
Hemoglobin level, g/dL	9.7 (9.05–11.5)	6.3 (5.3–7.7)	9.6 (8–10.15)	.0002
Platelet level, platelets/μL	494 (320.5–655.5)	93 (76–184)	419 (273–605)	.0021
CSF WBC count, cells/μL	228 (34–1000)	1 (0–2)	2 (0–2)	.0003
CSF protein level, mg/dL	1.865 (0.875–2.645)	0.24 (0.205–0.375)	0.195 (0.165–0.24)	.0001
Blood glucose, mg/dL	5.9 (3.8–7.1)	4.6 (3.75–5.05)	4.5 (3.85–6.65)	.4281
CSF glucose, mg/dL	0.95 (0.6–2.15)	3.4 (3–3.7)	3.3 (2.85–3.9)	.0004
Ratio of CSF to blood glucose	0.19 (0.12–0.41)	0.72 (0.65–0.96)	0.70 (0.61–0.77)	.0011
Plasma HRP2 level	0.0025 (0–0.006)	6.1915 (0.4845–13.3285)	0 (0–0.2235)	.0001
CSF HRP2 level	0.002 (0–0.0035)	0.0265 (0.0085–0.108)	0.003 (0.001–0.01)	.0002
Hospitalization duration, d	9.5 (8.5–14)	3 (2.5–3.5)	4 (2.5–6)	.0003

Data are median (interquartile range).

Abbreviations: CSF, cerebrospinal fluid; HRP2, histidine-rich protein 2; iRBC, infected red blood cell; WBC, white blood cell.

^a^ By the Kruskal-Wallis test.

### Protein Separation by 2-Dimensional Gel Electrophoresis

**Figure 1.**
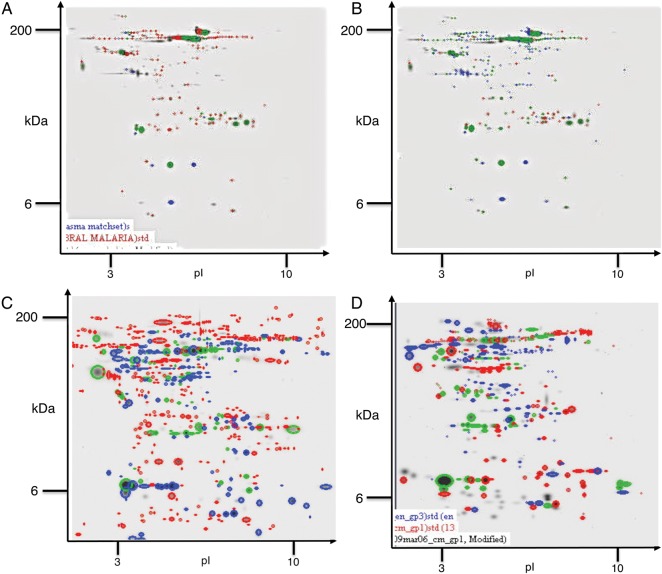
Composite gel showing the differences between gels in (*A*) plasma collected from children with a diagnosis of cerebral malaria (CM) and acute bacterial meningitis (ABM; *A*), plasma collected from children with a diagnosis of CM and nonspecific encephalopathy (*B*), cerebrospinal fluid (CSF) collected from children with a diagnosis of CM and ABM (*C*), and CSF collected from children with a diagnosis of CM and ABM (*D*). Composite gel maps were created using the comparison tool in PDQuest. The gel maps were created by analyzing duplicate gels for 12 patients’ biological replicates (2 gels per patient sample). The extent of the correlation of protein spots between the individual gels was >0.77 (a coefficient of 1.00 indicates that the replicate gels are perfectly similar). Red spots are spots found in the CM gel map, blue spots are unique to the comparator, and green spots depict spots found in both.

Reference gels for each phenotype were created using PDQuest. For plasma, averages of 200, 194, and 71 spots were included in the cerebral malaria, nonspecific encephalopathies, and acute bacterial meningitis gels, respectively. For CSF, averages of 150 spots were included in the cerebral malaria and nonspecific encephalopathies reference gels, and 80 spots were included in the acute bacterial meningitis gel. The 2-dimensional gel electrophoresis patterns of both plasma and CSF samples showed significant differences between the 3 clinical phenotypes studied (Figure 1). Proteins of interest that were definitively identified using mass spectrometry are listed in Table 2 (plasma) and Table 3 (CSF). Plasma proteins identified were mainly involved in platelet activation and aggregation, protein transport, endocytosis and cell communication, lipid metabolism, and binding and protein/antigen/nucleotide binding. CSF proteins of interest were mainly involved in apoptosis and proteolysis.

**Table 2.** Host Proteins Identified as Differentially Expressed When Gels of Plasma From Individuals With Cerebral Malaria Were Compared to Other Phenotypes

Biological Process, Accession No.	Description	Spot Difference	
		Acute Bacterial Meningitis	Nonspecific Encephalopathy
Platelet activation/aggregation			
P01009	Alpha-1-antitrypsin	Unique	Unique
P02647	Apolipoprotein A	…	Down
P02787	Serotransferrin precursor	Unique	Missing
P02787	C chain of human serum transferrin	…	Missing
P02679	F chain of fragment D of fibrinogen	…	Missing
P52735	Guanine nucleotide exchange factor VAV2	Unique	Unique
D3DP16	Fibrinogen γ chain, isoform CRA_a	…	Missing
Protein transport/endocytosis/cell communication			
O60493	Sorting nexin-3 (protein SDP3)	Down	Down
P19652	Alpha-1-acid glycoprotein, type 2	Up	Up
P02763	Alpha-1-acid glycoprotein, type 1	Up	Up
P02768	Albumin, isoform CRA_g	…	Unique
Lipid metabolism and binding			
P02647	A chain of human apolipoprotein A-I	…	Down
Protein/antigen/nucleotide binding			
Q96Q89	M-phase phosphoprotein 1	Up	Unique
P16871	Interleukin 7 receptor, isoform CRA_b	…	Missing

**Table 3.** Host Proteins Identified as Differentially Expressed When Gels of Cerebrospinal Fluid From Individuals With Cerebral Malaria Were Compared to Other Phenotypes

UniProt KB Accession No.	Description	Spot Difference		Gene Ontology
		Acute Bacterial Meningitis	Nonspecific Encephalopathy	
P02766	Transthyretin precursor	…	Unique	Protein transport/hormone activity and binding
P62745	ρ-related GTP-binding protein	Down	…	Angiogenesis/apoptosis/cell differentiations
P50213	Isocitrate dehydrogenase [NAD] subunit α, mitochondrial precursor	Unique	…	Carbohydrate metabolic processing
Q9NRR2	Tryptase γ preproprotein	Down	…	Proteolysis
O60423	Probable phospholipid-transporting ATPase IK	Unique	…	Cation transport
A4D1V4	39S ribosomal protein L32	Unique	…	Translation

### Protein Separation by 2-Dimensional LC-MS/MS

**Figure 2.**
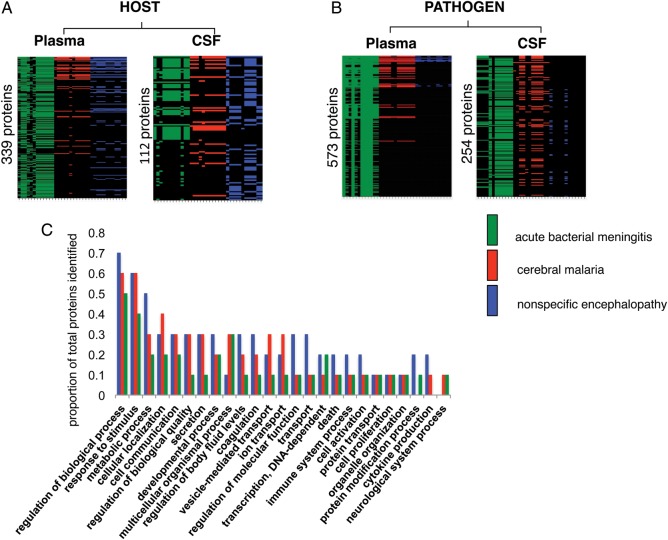
Heat maps showing distribution of proteins expressed using a 2-dimensional liquid chromatography tandem mass spectrometry strategy. *A*, Host proteins in plasma and cerebrospinal fluid (CSF). *B*, *Plasmodium falciparum* proteins in plasma and CSF. *C*, Graph showing breakdown of the gene ontology categorization of host proteins identified in plasma.

Analysis of plasma samples using 2-dimensional LC-MS/MS revealed a total of 339 host proteins and 573 falciparum proteins. In CSF, we identified 113 host proteins and 254 falciparum proteins. Heat maps of all proteins identified in all 36 patients are shown in Figure 2.

**Figure 3.**
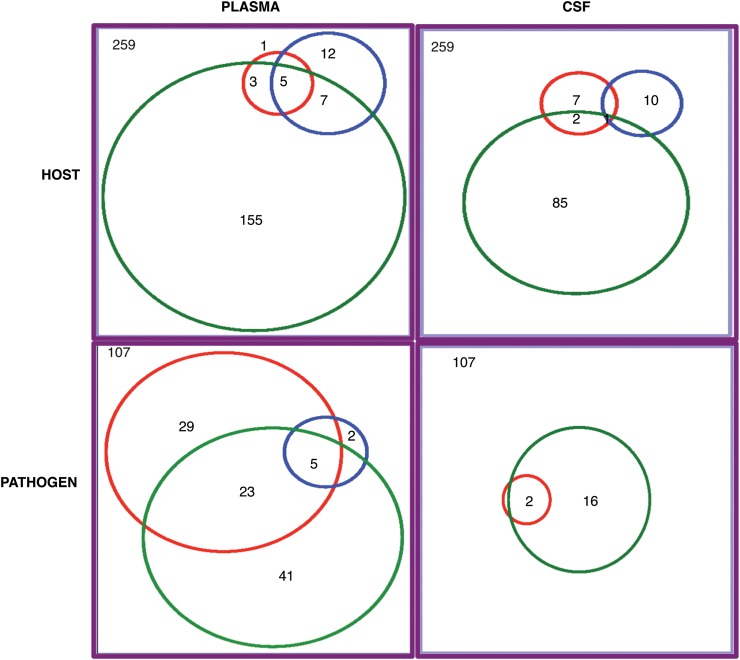
Venn diagram showing distribution of proteins of interest expressed using a 2-dimensional liquid chromatography tandem mass spectrometry strategy. The red circles represent cerebral malaria, the green circles represent acute bacterial meningitis, and the blue circles represent nonspecific encephalopathy. Abbreviation: CSF, cerebrospinal fluid.

We selected 259 host proteins of interest (Figure 3 and Supplementary Table 1). Selection was based on whether the protein was of known function and unique to the cerebral malaria phenotype in plasma (n = 1) or CSF (n = 7) or whether it was identified in the other 2 phenotypes but not in cerebral malaria. Seventy-six proteins were found in CSF but not in plasma, and 29 were found in both plasma and CSF. Of particular interest were 3 brain-specific proteins found in plasma namely brain-specific angiogenesis inhibitor 2, calcium/calmodulin-dependent protein kinase IV in acute bacterial meningitis, and spectrin nonerythroid β chain 3 in cerebral malaria. In addition, we also identified 14 other proteins that have been implicated in cerebral malaria or other brain injury (Table 4).

**Table 4.** Host Proteins of Interest Implicated in Brain Injury Identified Using a 2-Dimensional Liquid Chromatography Tandem Mass Spectrometry Strategy

Protein	UniProt KB Accession No.	CSF	Plasma	Reference(s)
		CM; NE; ABM	CM; NE; ABM	
Cytoplasmic protein NCK2	O43639	Yes; No; Yes	No; No; Yes	[29]
Brain-specific angiogenesis inhibitor 2	O60241	No; No; No	No; No; Yes	[30]
Spectrin beta chain, erythrocyte	P11277	No; No; No	No; No; Yes	[31, 32]
Secretogranin-2 precursor	P13521	Yes; No; No	No; No; Yes	[33]
Sodium/glucose cotransporter 1	P13866	Yes; No; No	No; No; No	[34, 35]
Vinculin	P18206	No; Yes; No	No; No; No	[36]
Neurofibromin	P21359	No; No; Yes	No; No; No	[37, 38]
Alanine glyoxylate aminotransferase	P21549	Yes; No; No	No; No; No	[39]
Retinol-binding protein 2	P50120	No; No; No	No; Yes; yes	[5, 40]
Retinal guanylyl cyclase 2 precursor	P51841	Yes; No; No	Yes; No; Yes	[19, 41]
Neurexophilin-1 precursor	P58417	No; No; No	No; No; Yes	[42]
Calcium/calmodulin-dependent protein kinase IV; brain Ca	Q16566	No; No; No	No; No; Yes	[41, 43]
phospholipase C, β 2	Q59F77	Yes; No; No	No; No; No	[44]
Spectrin β chain, brain 3	Q9H254	Yes; No; No	Yes; No; No	[15]
Reticulon 4	Q9NQC3	No; No; No	No; No; Yes	[45, 46]
Bromodomain adjacent to zinc finger domain protein 2B	Q9UIF8	Yes; No; Yes	No; No; Yes	[47]
NDRG2 protein	Q9UN36	No; Yes; No	Yes; No; No	[48, 49]
Endothelial lipase precursor	Q9Y5X9	Yes; No; No	No; No; No	[50]

Abbreviations: ABM, acute bacterial meningitis; CM, cerebral malaria; CSF, cerebrospinal fluid; NE, nonspecific encephalopathies.

In total, we compiled a list of 107 nonhypothetical *P. falciparum* proteins of interest (Figure 3 and Supplementary Table 2) that were found in plasma and CSF. This list included proteins involved in host cell modification, such as heat shock protein 40; in antigenic variation and host cell interaction, such as 17 variants of erythrocyte membrane protein 1; and 7 rifins.

We wondered whether the presence of parasite proteins in slide-negative children could be due to a recent infection, and we therefore measured levels of *p*HRP2, a parasite protein that has been shown to have a half-life of about 2 weeks after infection [10]. All children with cerebral malaria had *p*HRP2 levels in plasma and CSF that were above set cutoff levels. One child with acute bacterial meningitis and 3 children with nonspecific encephalopathy had high levels in plasma. The 3 children with nonspecific encephalopathy also had levels of *p*HRP2 in CSF that were equal to or above the set cutoff level. Interestingly, 3 children with acute bacterial meningitis had levels of *p*HRP2 in CSF equal to or above the set cutoff level but did not have any *p*HRP2 detected in the plasma.

## DISCUSSION

Pathogenic states in children with impaired consciousness in malaria-endemic areas could be reflected by changes in protein biomarkers in both plasma and CSF. Proteomic approaches allow for the analysis of large numbers of proteins at the same time, and this may help elucidate pathways that can be targeted for therapy. Additionally, proteomic platforms allow for the measurement of low-abundance proteins in complex materials such as plasma. This article describes the use of proteomic platforms to elucidate differences in plasma and CSF proteomes collected from children in a malaria-endemic area presenting with impaired consciousness. In this analysis, by use of 2 different proteomic strategies, 259 host proteins and 107 parasite (ie, *P. falciparum)* proteins were identified as differentially expressed in both plasma and CSF, and these could help elaborate mechanistic differences in the different encephalopathies describe here. Importantly, some of the proteins could help point to the cause of disease in the group of patients with nondefined encephalopathy.

The first striking observation was the presence of *P. falciparum* proteins in both plasma and CSF of slide-negative children with acute bacterial meningitis. This result raises 2 possibilities: (1) the children have had a recent episode of malaria, which could predispose them to acute bacterial meningitis; and (2) children in malaria-endemic areas have persistently low levels of parasite proteins in their sera, although one wonders why fewer proteins were identified in the slide-negative children without acute bacterial meningitis. To confirm the second point, we would need to analyze samples from healthy community controls. The presence of pathogen proteins in CSF could be due to a leaky blood brain barrier. However, not all proteins found in the CSF were found in plasma, and additionally we did not always find a correlation with plasma *p*HRP2 levels and CSF *p*HRP2 levels. We therefore cannot rule out that proteins in the CSF are there as a result of sequestration in the brain and/or leukocyte migration into the brain.

We found 14 spots differentially regulated based on gels from the cerebral malaria group, compared with gels from the other 2 groups. Seven of these were identified as proteins that play a role in platelet activation and aggregation (Table 2). In addition, using the 2-dimensional LC-MS/MS strategy, we identified 13 proteins that play a role in coagulation and that were missing in plasma from the cerebral malaria group (Supplementary Table 1) or were found in CSF from acute bacterial meningitis group and not in the other groups. This is not surprising because thrombocytopenia is associated with cerebral malaria [11, 12], and in fact children in this study had lower levels of platelets. Parasite platelet complexes could be a cause of reduced platelet counts, and a proteomic study of platelet microparticles could help clarify mechanisms leading to thrombocytopenia.

Differentially expressed proteins identified in this study, such as α 1 acid glycoprotein type 1 and type 2 (orosomucoid 2), reticulon 4, and retinol binding protein 2 could be markers of an uncontrolled and harmful inflammatory response. An increase in orosomucoid 2, differentially expressed in both plasma and CSF of patients with cerebral malaria, together with an increased production of ceruloplasmin and glutathione, would enhance antioxidant defenses and limit the stimulatory effects of oxidant molecules on cytokine production. This acute-phase protein has previously been studied in connection with malaria [13], and it has been suggested that its production may reflect the severity of the acute phase response [14].

Spectrin β chain brain 3, a protein that belongs to a family of spectrin proteins, was differentially expressed in both plasma and CSF of children with cerebral malaria. In our previous mouse study [5], this protein was differentially expressed in plasma of mice infected with *Plasmodium berghei*, compared with expression in noninfected mice. In the brain, this protein is enriched in myelinated neurons, where it colocalizes with ankyrin at axon initial segments and nodes of Ranvier and participates in the clustering of voltage-gated Na^+^ channels and cell-adhesion molecules at initial segments and nodes of Ranvier [15]. Additionally, spectrin proteins have also been identified as binding partners for various *P. falciparum* proteins. In particular, spectrins have been shown to associate with heat shock protein 40 [16], and in ring-stage infected red blood cells (RBCs), ring-infected erythrocyte surface antigen associates with spectrin and stabilizes the membrane skeleton. In mature-stage parasitized RBCs, knob-associated His-rich protein molecules self-associate to form conical structures that interact with spectrin. Pf332 and mature-parasite-infected erythrocyte surface antigen bind to the junction complex, while *P. falciparum* erythrocyte membrane protein 3, identified in this study in plasma of children with cerebral malaria and acute bacterial meningitis, binds to spectrin, further compromising RBC membrane deformability [17]. These interactions stabilize spectrin tetramers in the infected RBC, increasing resistance to further parasite invasion of the cell by increasing infected RBC rigidity, and could facilitate sequestration and inhibit splenic clearance [18]. We hypothesize that the difference in spectrin expression in our study could be linked to these interactions with the infected RBC.

Excitotoxic cell death in CNS disorders is partly due to dysfunction of the sodium/potassium pump, resulting in an increased uptake of water, which could as a consequence lead to an increased influx of calcium. Retinal guanylyl cyclase 2 precursor is a gene that displays calcium-dependent regulation [19]. In addition, sodium-dependent glucose transport could also be affected, and this could result in the differential expression of sodium/glucose cotransporter 1 (SGLT1) seen in the CSF of patients with cerebral malaria. SGLT1 has been shown to be expressed in neurons and is upregulated during metabolic stress when there is a decrease in D-glucose content [20]. The sorting nexin family of proteins, which contain a Phox homology domain, play crucial roles in regulating the intracellular membrane trafficking of the endocytic pathway [21]. SNX3, which was differentially expressed in this study, is associated with the early endosome through the PX domain, which is capable of interaction with phosphatidylinositol-3-phosphate. Overexpression of SNX3 alters endosomal morphology and delays transport to the lysosome.

It is important to consider potential limitations of our study design and analysis strategy. First, proteomic studies require careful sample collection and storage. Prolonged contact of CSF and plasma with cellular components has been shown to affect protein and peptide quality because of the presence of proteolytic enzymes in plasma [22, 23] and CSF [24, 25]. Samples analyzed in this study were centrifuged within an hour of collection to remove all cellular components, although protease inhibitors, which can minimize degradation, were not added. These inhibitors can interfere with peptide and amino acid MS signals [26], and some studies suggest that there are no differences in CSF and plasma proteomes determined in the presence or absence of protease inhibitors [27, 28]. Second, a “normal” control could not be established for this study because CSF can only be ethically obtained from children with impaired consciousness. However, future studies could include a nonfebrile group of children with impaired consciousness, such as those with epileptic seizures or poisoning or those who have a lumbar puncture to rule out meningitis. Third, the samples were obtained at admission, and therefore the effects of antimalarial treatment and other effects of disease progression, such as the inflammatory responses that may influence the protein levels, could not be fully assessed. Fourth, cytokines and chemokines that have previously been identified as playing a role in cerebral malaria were not differentially expressed in our study. Two possible reasons for this finding are that (1) semiquantitative 2-dimensional gel analysis may not be sensitive enough to detect such low abundant proteins and that (2) the 2-dimensional LC-MS/MS method described in this study was not quantitative and therefore only reported the presence or absence of proteins in a patient group. Most cytokines and chemokines implicated in malaria were present in all 3 groups.

Despite the limitations mentioned above, the results from this study show that novel disease specific biomarkers can be identified using proteomic strategies. In addition to providing insight into underlying pathophysiological mechanisms, they have the potential after thorough validation to be used as biomarker panels, which may be of value in the early diagnosis of disease and in monitoring responses to therapies. In particular, this study has shown that there are proteins uniquely expressed in cerebral malaria. The next phase of this work will be to rigorously assess and validate the diagnostic value of these differentially expressed proteins before transfer to a suitable platform that can generate an affordable point-of-care diagnostic.

## Supplementary Data

Supplementary materials are available at *The Journal of Infectious Diseases* online (http://jid.oxfordjournals.org/). Supplementary materials consist of data provided by the author that are published to benefit the reader. The posted materials are not copyedited. The contents of all supplementary data are the sole responsibility of the authors. Questions or messages regarding errors should be addressed to the author.

Supplementary Data
