# Supplementary material for: Plasma and Cerebrospinal Proteomes From Children With Cerebral Malaria Differ From Those of Children With Other Encephalopathies
Source: J Infect Dis. 2013 Jul 25;208(9):1494–503. doi: 10.1093/infdis/jit334 (PMC3789566; doi:10.1093/infdis/jit334)
Supplement: Supplementary Data [file supp_208_9_1494__index.html]

Plasma And Cerebrospinal Proteomes Of Children With Cerebral Malaria Differ From Children With Other Encephalopathies — Plasma and Cerebrospinal Proteomes From Children With Cerebral Malaria Differ From Those of Children With Other Encephalopathies — Plasma and Cerebrospinal Proteomes From Children With Cerebral Malaria Differ From Those of Children With Other Encephalopathies — Supplementary Data 

# Plasma and Cerebrospinal Proteomes From Children With Cerebral Malaria Differ From Those of Children With Other Encephalopathies

## Supplementary Data

Supplementary Data

**Files in this Data Supplement:**

- Supplementary Data - Docx file
- Supplementary Table 1 - docx file
- Supplementary Table 2 - docx file
