# Supplementary material for: Plasma and Cerebrospinal Proteomes From Children With Cerebral Malaria Differ From Those of Children With Other Encephalopathies
Source: J Infect Dis. 2013 Jul 25;208(9):1494–503. doi: 10.1093/infdis/jit334 (PMC3789566; doi:10.1093/infdis/jit334)
Supplement: Supplementary Data [file supp_jit334_jit334supp.docx]

**Supplementary Methods**

**In-gel Trypsin Digestion**

For plasma, the protein spots of interest were excised from Coomassie stained gels and transferred to the eppendorf tubes. The chopped gel pieces were washed in 50% acetonitrile/ 25mM ammonium bicarbonate (ABC), pH 7.8, and dried in a vacuum concentrator. 4-10 μl digestion buffer (10 μg/ml modified sequencing grade trypsin (Sigma-Aldrich) in 25 mM ABC) was added to the dried gel pieces and incubated overnight at 37°C. Resulting peptides were extracted by addition of 4μl water followed by 7μl of 30% acetonitirile/0.1% TFA followed by vortexing and brief centrifugation. The supernatant was transferred to a clean tube and vacuum concentrated to approximately 5μl.

For CSF, spots cut from silver stained gels were digested using a modified method described by [[1](#_ENREF_1)]. The silver stained spots were washed twice in 50% acetornitrile in 100mM ABC for five minutes. The spots were then dehydrated using 100% acetonitrile until they turned opaque white, followed by evaporation of excess acetonitrile in a vacuum concentrator. Digestion buffer (5-10µl of 10µg/ml modified sequencing grade trypsin (Sigma-Aldrich) in 25mM ABC), was added to the dried gel pieces and incubated overnight at 37˚C. Resulting peptides were extracted by first incubating the digest in 7µl of distilled water and then adding 30% acetonitrile in 0.1% TFA and sonicating for 30 minutes. The digest was then vortex mixed and centrifuged for 2 minutes. The supernatant was transferred and vacuum concentrated to about 2µl.

##### **In-Solution Trypsin Digestion**

Samples were reduced in 3µl of 0.01M DTT in water for every 10µg of protein. This was done for 30 minutes at 30˚C. To prevent the disulphide bonds from reforming, samples were then alkylated using 3µl of 0.01M IAA in 50mM NH_4_HCO_3_ for every 10µg of protein and incubated for another 30 minutes at room temperature (~20°C-22°C). To quench any excess IAA an excess of the 0.01M DTT was added to the solution. 1µg (20µg/ml) of trypsin was added for every 100µg of protein to the sample and incubated overnight at 37˚C.

#### MALDI-ToF Mass Spectrometry

MALDI peptide mass fingerprinting was carried out using a AXIMA CFR Plus, (Shimadzu Biotech-Kratos Analytical, Manchester UK) operating in positive ion reflectron mode at an accelerating voltage of 20kV. The spectra were externally calibrated using a peptide mixture (Sigma, St Louis, MO) with masses 757.39 (Bradykinin), 1046.54 (Angiotensin II) and 2465.19 (ACTH). Protein identification was performed using the MASCOT® search engine (Matrix Science, London, UK).

#### ESI-Ion Trap Tandem Mass Spectrometry

Tandem mass spectrometry was carried out on a LCQ Deca XP Plus ion trap mass spectrometer (ThermoFinnigan, USA) equipped with an electrospray source. The LCQ Deca XP Plus was tuned using Angiotensin I (singly charges at m/z 1296.7, doubly charged at m/z 648.8 and triply charged at m/z 432.9) and calibrated according to the manufacturers’ instructions. To maintain a stable spray, the spray voltage was adjusted to (1.5-1.8kV). The capillary temperature was set at 250˚C and the normalised collision energies were set at 35% for MS/MS. The mass spectrometer was operated on a data-dependent “triple play” mode where the 3 most intense ions in the full scan were subjected to a zoom scan followed by MS/MS. Dynamic exclusion set at 0.5 min was used to obtain MS/MS spectra from any co-eluting peptides.

The spectra were evaluated using the TurboSEQUEST® algorithm in BioWorks ™v 3.1 software provided by ThermoFinnigan.

**Mascot® Search Parameters**

Mass lists generated from the MALDI –ToF were searched against the NCBInr database. All searches were performed with the search parameters set as: Database: NCBInr, Taxonomy: *Homo Sapiens* or *Plasmodium Falciparum*, Enzyme: Trypsin, Tolerance: ±0.2Da, Fixed Modifications: Carbamidomethyl (C), Variable Modifications: Oxidation of methionine, Charge State MH+, Maximum missed cleavages: 1.

**TurboSEQUEST® Search Parameters**

MS/MS spectra were searched against the Human and *P. falciparum* subsets of the NCBInr database and the *P. falciparum* database (PlasmoDB version 4.4) downloaded from the Sanger Institute. All searches were performed with the search parameters set as: Modifications: Carbamidomethyl (C), Variable Modifications: Oxidation of methionine, Charge State MH+, Maximum missed cleavages = 1. Searches were then repeated on the entire NCBInr database and only proteins identified from both searches were accepted. In addition, proteins were accepted as legitimate identifications if they had two or more peptides with cross-correlation score (Xcorr) of >1.5 for singly charged ions, >2.00 for doubly charged ions and >2.50 for triply charged ions each with a delta correlation (DelCn) >0.1.

**1. Terry DE, Umstot E, Desiderio DM. Optimized sample-processing time and peptide recovery for the mass spectrometric analysis of protein digests. J Am Soc Mass Spectrom 2004; 15:784-94.**
