# Supplementary material for: Plasma and Cerebrospinal Proteomes From Children With Cerebral Malaria Differ From Those of Children With Other Encephalopathies
Source: J Infect Dis. 2013 Jul 25;208(9):1494–503. doi: 10.1093/infdis/jit334 (PMC3789566; doi:10.1093/infdis/jit334)
Supplement: Supplementary Data [file supp_jit334_jit334supp_table1.docx]

| **SUPPLEMENTARY TABLE 1. Host Proteins of Interest** | |  |  |
| --- | --- | --- | --- |
| **Protein family; UniProt_KB ACCESSION** | **Protein Name** | **CSF_CM;NE;ABM** | **Plasma_CM;NE;ABM** |
| ***Metalloproteases;peptidases; proteolytic enzymes*** |  |  |  |
| P50281 | matrix metalloproteinase 14 preproprotein | no;no;no | no;no;yes |
| Q99797 | mitochondrial intermediate peptidase | no;no;no | no;no;yes |
| O00291 | Huntingtin-interacting protein 1 | no;no;no | no;yes;yes |
| P08311 | A Chain A, Cathepsin G | no;no;yes | no;no;no |
| P23276 | Kell blood group glycoprotein | no;no;yes | no;no;no |
| Q15418 | Ribosomal protein S6 kinase alpha-1 | no;no;no | no;yes;no |
| P24158 | Myeloblastin precursor | no;no;yes | no;no;no |
| P25787 | proteasome alpha 2 subunit; proteasome subunit HC3; proteasome componen | no;no;no | no;yes;no |
| P40818 | Ubiquitin carboxyl-terminal hydrolase 8 | no;no;no | no;no;yes |
| Q14868 | Effector cell proteinase receptor 1 | no;no;yes | no;no;no |
| Q59E93 | membrane alanine aminopeptidase precursor | no;no;no | no;no;yes |
| Q9UGI0 | Ubiquitin thioesterase ZRANB1 | no;yes;no | no;no;no |
| Q9UKL3 | CASP8-associated protein 2 | no;no;yes | yes;yes;yes |
| Q9Y4W6 | AFG3 ATPase family gene 3-like 2 | no;no;no | no;no;yes |
| P10619 | Lysosomal protective protein precursor | no;no;yes | no;no;no |
| Q9Y5X9 | Endothelial lipase precursor | yes;no;no | no;no;no |
| Q9Y5T5 | ubiquitin specific protease 16 | no;no;no | no;no;yes |
| ***ADC-like; P-loop containing nucleoside triphosphate hydrolases; NADH dehydrogenases; Glyceraldehyde-3-phosphate dehydrogenase-like*** |  |  |  |
| O75380 | NADH dehydrogenase [ubiquinone] iron-sulfur protein 6 | no;no;yes | no;no;no |
| O75489 | NADH dehydrogenase [ubiquinone] iron-sulfur protein 3 | no;no;no | no;no;yes |
| P11413 | Glucose-6-phosphate 1-dehydrogenase | no;no;no | no;no;yes |
| P17568 | NADH dehydrogenase [ubiquinone] 1 beta subcomplex subunit 7 | no;no;no | no;no;yes |
| P28845 | Corticosteroid 11-beta-dehydrogenase isozyme 1 | no;no;yes | no;no;no |
| Q00796 | Sorbitol dehydrogenase | no;no;no | no;no;yes |
| P46459 | Vesicle-fusing ATPase | no;no;no | no;no;yes |
| ***alpha-catenin/vinculin-like*** |  |  |  |
| P18206* | Vinculin | no;yes;no | no;no;no |
| ***Anaphylotoxins (complement system); Terpenoid cyclases/Protein prenyltransferases; Alpha-macroglobulin receptor domain*** |  |  |  |
| P01023 | alpha 2 macroglobulin precursor | no;no;yes | yes;yes;yes |
| P01024 | complement component 3 precursor | no;no;yes | no;no;no |
| ***Anticodon-binding domain of a subclass of class I aminoacyl-tRNA synthetases; ValRS/IleRS/LeuRS editing domain; Nucleotidylyl transferase*** |  |  |  |
| Q15031 | Probable leucine--tRNA ligase | no;no;yes | no;no;no |
| ***Concanavalin A-like lectins/glucanases; B-box zinc-binding domain*** |  |  |  |
| Q9BZY9 | E3 ubiquitin-protein ligase TRIM31 | no;no;no | no;no;yes |
| ***SH3-domain; PH domain-like; SH2 domain;DH-domain*** |  |  |  |
| O43639 | Cytoplasmic protein NCK2 | yes;no;yes | no;no;yes |
| P52735* | Guanine nucleotide exchange factor VAV2 | no;no;yes | no;no;no |
| Q9UNA1 | Rho GTPase-activating protein 26 | no;no;no | no;no;yes |
| Q5JY90 | Bruton agammaglobulinemia tyrosine kinase | no;no;yes | no;no;no |
| ***Calcium ion binding domains*** |  |  |  |
| P05109 | S100 calciumbinding protein A8 | no;no;yes | no;no;no |
| P98194 | Calcium-transporting ATPase type 2C member 1 | no;no;no | no;no;yes |
| P06702 | S100 calcium binding protein A9 | no;no;yes | no;no;no |
| O75747 | phosphoinositide-3-kinase, class 2, gamma polypep | no;no;no | no;no;yes |
| P46939 | utrophin | no;no;yes | no;no;no |
| P98194 | Calcium-transporting ATPase type 2C member 1 | no;no;no | no;no;yes |
| Q16853 | copper containing amine oxidase 3 precursor | no;no;yes | no;no;no |
| Q14416 | Glutamate receptor, metabotropic 2 precursor | no;no;no | no;no;yes |
| Q16853 | copper containing amine oxidase 3 precursor | no;no;yes | no;no;no |
| Q4V347 | Allograft inflammatory factor 1 | no;no;yes | no;no;no |
| Q93034 | Vasopressin-activated calcium-mobilizing receptor 1 | no;no;yes | no;no;yes |
| O43745 | Calcineurin B homologous protein 2 | no;no;no | no;no;yes |
| P78504 | jagged 1 precursor | no;no;yes | no;no;no |
| Q14571 | Inositol 1,4,5-triphosphate receptor, type 2 | no;no;no | no;no;yes |
| Q14574 | Desmocollin-3 precursor | no;no;no | no;no;yes |
| Q59F77 | phospholipase C, beta 2 | yes;no;no | no;no;no |
| Q8WZ42 | Titin | no;no;yes | no;no;yes |
| Q9NZ20 | Group 3 secretory phospholipase A2 precursor | no;no;no | no;no;yes |
| ***Formyltransferase*** |  |  |  |
| P22102 | Phosphoribosylglycinamide formyltransferase | no;no;no | no;no;yes |
| ***Zinc Fingers*** |  |  |  |
| P15622 | Zinc finger protein 250 | no;no;yes | no;no;no |
| P35789 | Zinc finger protein 93 | no;no;yes | no;yes;no |
| Q14839 | chromodomain helicase DNA binding protein 4 | no;no;no | no;no;yes |
| Q15075 | Early endosome antigen 1 | no;no;no | no;no;yes |
| ***Galactose-binding domain-like; Coagulation factor VIII, membrane-binding peptide*** |  |  |  |
| P00451* | Coagulation factor VIII, procoagulant component, isoform a precursor; | no;no;no | no;no;yes |
| ***Globin-like; Immunoglobulin; MHC antigen-recognition domain*** |  |  |  |
| P02042* | Delta globin | yes;yes;yes | no;yes;no |
| ***Spectrin repeat; Calponin-homology domain, CH-domain; PH domain-like; PX domain*** |  |  |  |
| P11277 | Spectrin beta chain, erythrocyte | no;no;no | no;no;yes |
| Q9H254 | Spectrin beta chain, brain 3 | yes;no;no | yes;yes;yes |
| Q9Y4H2 | Insulin receptor substrate 2 | no;no;no | no;yes;yes |
| Q13596 | sorting nexin 1 isoform a | no;no;no | no;no;yes |
| ***Lipocalins*** |  |  |  |
| P50120 | Retinol-binding protein 2 | no;no;no | no;yes;yes |
| P02763 | Alpha-1-acid glycoprotein 1 precursor | no;no;yes | yes;no;no |
| P19652 | Alpha-1-acid glycoprotein 2 precursor | no;no;yes | no;yes;no |
| Q01469 | fatty acid binding protein 5 | no;no;no | no;no;yes |
| ***NagB/RpiA/CoA transferase-like*** |  |  |  |
| P46926 | Glucosamine-6-phosphate isomerase 1 | no;no;no | no;no;yes |
| P55809 | Succinyl-CoA:3-ketoacid-coenzyme A transferase 1 | no;no;yes | no;no;no |
| ***P-loop containing nucleoside triphosphate hydrolases*** |  |  |  |
| O00338 | Sulfotransferase 1C2 | no;yes;no | no;no;no |
| P49888 | Estrogen sulfotransferase | no;no;yes | no;no;no |
| P51157 | Ras-related protein Rab-28 precurso | no;no;no | no;no;yes |
| P52848 | Bifunctional heparan sulfate N-deacetylase/N-sulfotransferase 1 | no;no;yes | no;no;no |
| Q99661* | Mitotic centromere-associated kinesin | no;no;no | no;no;yes |
| ***Periplasmic binding protein-like II; Antimicrobial beta-hairpin*** |  |  |  |
| P02788 | Lactotransferrin precursor | no;no;yes | no;no;no |
| P51841 | Retinal guanylyl cyclase 2 precursor | yes;no;no | yes;no;yes |
| ***Ribosome and ribosomal fragments*** |  |  |  |
| P62910 | 60S ribosomal protein L32 | no;no;no | no;no;yes |
| P83731 | 60S ribosomal protein L24 | no;no;no | no;no;yes |
| ***Serpins; Angiotensin*** |  |  |  |
| P01011 | Alpha-1-antichymotrypsin precursor | no;no;yes | no;no;no |
| Q9UIV8 | Serpin B13 | no;no;no | no;no;yes |
| ***nucleases;transferases;lipases;kinases;phosphatases;hydrolases*** |  |  |  |
| O14578 | Citron Rho-interacting kinase | no;no;no | no;no;yes |
| O14920 | Inhibitor of nuclear factor kappa-B kinase subunit beta | no;no;no | no;no;yes |
| P78347 | General transcription factor II-I | no;no;no | no;no;yes |
| Q16566 | Calcium/Calmodulin-dependent protein kinase IV; brain Ca | no;no;no | no;no;yes |
| O75962 | Triple functional domain protein | no;no;no | no;no;yes |
| O96017 | protein kinase CHK2 isoform a | no;no;no | no;no;yes |
| P34925 | Tyrosine-protein kinase RYK precursor | no;yes;no | no;no;no |
| P42345 | Serine/threonine-protein kinase | no;no;yes | no;no;no |
| P78527 | DNA-activated protein kinase | no;no;no | no;no;yes |
| Q13233 | Mitogen-activated protein kinase kinase kinase 1 | no;no;no | no;no;yes |
| Q60FE5 | Filamin A | no;no;no | no;no;yes |
| Q6P2N0 | myosin light chain kinase isoform 1 | no;no;no | no;no;yes |
| Q9H2X6 | Homeodomain-interacting protein kinase 2 | no;no;yes | no;no;no |
| Q9UDY2 | Zonula occludens 2 protein | no;no;yes | no;no;no |
| Q9ULW0 | Targeting protein for Xklp2 | no;yes;no | no;no;no |
| P51530 | DNA replication ATP-dependent helicase-like homolog | no;no;no | no;no;yes |
| Q15027 | centaurin beta1 | no;no;no | no;no;yes |
| Q96DT5 | Dynein heavy chain 11, axonemal | no;no;no | no;no;yes |
| P45844 | ATPbinding cassette, subfamily G, member 1 | no;no;yes | no;no;no |
| Q92539 | lipin 2 | no;no;yes | no;no;no |
| Q9NUT2 | ATP-binding cassette sub-family B member 8, mitochondrial precursor | no;no;no | no;no;yes |
| O94808 | glutamine-fructose-6-phosphate transaminase 2 | no;no;no | no;no;yes |
| P06737 | Glycogen phosphorylase (liver form) | no;no;no | no;yes;yes |
| P21549 | alanineglyoxylate aminotransferase | yes;no;no | no;no;no |
| P53396 | ATP-citrate synthase | no;yes;no | no;no;no |
| Q9UI43 | cell division protein FtsJ | no;no;yes | no;no;no |
| Q9Y4C5 | Carbohydrate sulfotransferase 2 | no;no;no | no;no;yes |
| P02647 | Apolipoprotein A1 | no;no;yes | no;yes;no |
| P06727 | Apolipoprotein A-IV precursor | no;no;no | no;no;yes |
| Q16772 | glutathione Stransferase A3 | no;no;yes | no;no;no |
| Q9NPF2 | chondroitin 4-sulfotransferase | no;no;no | no;no;yes |
| Q9UBR1 | betaureidopropionase | no;no;yes | no;no;yes |
| Q13867 | Bleomycin hydrolase | no;no;no | no;no;yes |
| Q93070 | ADP-ribosyltransferase 4 | no;no;no | no;no;yes |
| ***Transcription factors; DNA-binding domains*** |  |  |  |
| O15119 | T-box transcription factor TBX3 | no;no;no | no;no;yes |
| P04628 | Proto-oncogene Wnt-1 precursor | no;no;no | no;no;yes |
| P43694 | GATA binding protein 4 | no;no;no | no;no;yes |
| P48552 | Receptor interacting protein 140 | no;no;no | no;no;yes |
| Q13287 | N-myc and STAT interactor | no;yes;no | no;no;no |
| Q9UBB9 | tuftelin interacting protein 1 | no;no;no | no;no;yes |
| Q9UIV1 | CCR4-associated factor 1 | no;no;no | no;no;yes |
| O75376 | Nuclear receptor corepressor 1 | no;no;yes | no;no;no |
| O95600 | Krueppel-like factor 8 | no;no;no | no;no;yes |
| P49454 | Centromere protein F precursor | no;no;no | no;no;yes |
| P51523 | Zinc finger protein 84 | no;no;no | no;no;yes |
| Q03924 | zinc finger protein 117 | no;no;yes | no;yes;no |
| Q04917 | Protein 14-3-3 eta chain | no;no;no | no;no;yes |
| Q05481 | Zinc finger protein 91 | no;no;yes | no;yes;yes |
| Q10587 | Thyrotroph embryonic factor | no;no;yes | no;no;yes |
| Q15233 | Non-POU domain-containing octamer-binding protein | no;no;no | no;no;yes |
| Q15911 | Zinc finger homeobox protein 3 | no;no;no | no;no;yes |
| Q8WXB4 | Zinc finger protein 606 | no;no;no | no;yes;yes |
| Q92794 | Histone acetyltransferase KAT6A | no;no;no | no;no;yes |
| Q92833 | Jumonji protein | no;no;no | no;no;yes |
| Q96PE6 | zinc finger, imprinted 3 | no;no;no | no;no;yes |
| Q9H501 | chromosome 20 open reading frame 6 | no;no;yes | no;no;no |
| Q9UIF8 | Bromodomain adjacent to zinc finger domain protein 2B | yes;no;yes | no;no;yes |
| Q9UL36 | Zinc finger protein 236 | no;no;no | no;no;yes |
| Q9ULU4 | Protein kinase C-binding protein 1 | no;no;no | no;no;yes |
| Q9Y2V3 | retina and anterior neural fold homeobox | no;no;yes | no;no;no |
| Q9Y2X9 | Zinc finger protein 281 | no;no;yes | no;yes;no |
| Q9Y468 | Lethal(3)malignant brain tumor-like protein 1 | no;no;yes | no;no;no |
| P19338 | nucleolin | no;no;no | no;no;yes |
| P28370 | Probable global transcription activator SNF2L1 | no;no;yes | no;no;no |
| Q15424 | S64732 scaffold attachment factor B | no;no;no | no;no;yes |
| Q9HD90 | neurogenic differentiation 4 | no;no;no | no;no;yes |
| O60673 | DNA polymerase zeta catalytic subunit | no;no;no | no;no;yes |
| O95239* | Chromokinesin-A | no;no;yes | no;no;no |
| Q16544 | Homolog mismatch repair protein | no;no;no | no;yes;no |
| Q8TD26 | Chromodomain-helicase-DNA-binding protein 6 | no;no;no | no;no;yes |
| P17844 | DEAD/H (AspGluAlaAsp/His) box polypeptide 5 | no;no;yes | no;no;no |
| ***Signal Transducers*** |  |  |  |
| O60241 | Brain-specific angiogenesis inhibitor 2 | no;no;no | no;no;yes |
| O60658 | High affinity cAMP-specific and IBMX-insensitive 3',5'-cyclic phosphodiesterase 8A | no;no;yes | no;no;no |
| Q17R89 | Rho GTPase-activating protein 44 | no;no;no | no;no;yes |
| Q9Y271 | cysteinyl leukotriene receptor 1 | no;no;no | no;no;yes |
| Q9Y6K9 | NF-kappa-B essential modulator | no;no;no | no;no;yes |
| O00522 | Krev interaction trapped protein 1 | no;no;no | no;no;yes |
| P02671* | Fibrinogen alpha chain precursor | no;no;no | no;no;yes |
| P42331 | Rho GTPase-activating protein 25 | no;no;no | no;no;yes |
| P55196 | Afadin | no;no;no | no;no;yes |
| Q12955 | Ankyrin-3 | no;no;no | no;no;yes |
| Q52LW3 | PTPL1associated RhoGAP 1 | no;no;yes | no;no;no |
| Q9P2E9 | Ribosome binding protein 1 | no;no;yes | no;no;yes |
| P10809 | 60 kDa heat shock protein, mitochondrial precursor | no;no;yes | no;no;no |
| P36957 | Dihydrolipoamide succinyltransferase component of 2-oxoglutarate | no;no;no | no;no;yes |
| P02675* | Fibrinogen beta chain precursor | no;no;yes | no;no;no |
| ***Growth factors*** |  |  |  |
| P05019* | insulin-like growth factor 1 | no;no;no | no;no;yes |
| P11717 | Cation-independent mannose-6-phosphate receptor precursor | no;no;no | no;no;yes |
| Q9UN36 | NDRG2 protein | no;yes;no | yes;yes;yes |
| ***Calmodulin binding proteins*** |  |  |  |
| O95613 | Pericentrin | no;no;no | no;no;yes |
| P23634 | Plasma membrane calcium-transporting ATPase 4 | no;no;no | no;no;yes |
| P35580 | Cellular myosin heavy chain | no;no;yes | no;no;no |
| P35749 | smooth muscle myosin heavy chain 11, isoform SM1 | no;no;no | no;no;yes |
| Q13459 | myosin IXB | no;no;no | no;no;yes |
| ***Solute Carriers*** |  |  |  |
| O14975 | Solute carrier family 27 | no;no;no | no;no;yes |
| Q96BD0 | S21C_ Solute carrier family 21 member 12 (Sodium-independent organic ani | no;no;no | no;no;yes |
| Q9UP95 | Solute carrier family 12 member 4 | no;no;no | no;no;yes |
| ***Ion Channels*** |  |  |  |
| P37088 | Sodium channel, nonvoltage-gated 1 alpha | no;no;no | no;no;yes |
| Q92508 | Piezo-type mechanosensitive ion channel component 1 | no;no;no | no;yes;no |
| ***Others*** |  |  |  |
| O14617 | AP-3 complex subunit delta-1 | no;no;no | no;no;yes |
| O95292 | Vesicle-associated membrane protein -associated protein B and C | no;no;no | no;no;yes |
| O95456 | Proteasome assembly chaperone 1 | no;no;no | no;no;yes |
| P23219 | Prostaglandin G/H synthase 1 precursor | no;no;yes | yes;yes;yes |
| P35475 | alpha-L-iduronidase precursor | no;no;no | no;no;yes |
| P50876 | Probable E3 ubiquitin-protein ligase RNF144A | no;no;yes | no;no;no |
| P58417 | Neurexophilin-1 precursor | no;no;no | no;no;yes |
| P58743 | Prestin | no;no;yes | no;no;yes |
| Q05084 | Islet cell autoantigen 1 | no;no;no | no;no;yes |
| Q13367 | Adapterrelated protein complex 3 beta 2 subunit | no;no;yes | no;no;no |
| Q13451 | Peptidyl-prolyl cis-trans isomerase FKBP5 | no;no;yes | no;no;no |
| Q15582 | Transforming growth factor-beta-induced protein ig-h3 precursor | no;no;no | no;no;yes |
| Q93098 | Protein Wnt-8b precursor | no;yes;no | no;no;no |
| Q9H1J7 | Protein Wnt-5b precursor | no;no;no | no;no;yes |
| Q9H267 | vacuolar protein sorting 33B | no;no;no | no;no;yes |
| Q9Y3P9 | Rab GTPase-activating protein 1 | no;no;yes | no;no;no |
| B2RDF2 | pescadillo homolog 1, containing BRCT domain | no;no;yes | no;no;no |
| O15061 | Desmuslin | no;no;no | no;no;yes |
| O43155 | fibronectin leucine rich transmembrane protein 2 | no;no;no | no;no;yes |
| O43290 | U4/U6.U5 tri-snRNP-associated protein 1 | no;no;no | no;no;yes |
| O43516 | WAS/WASL-interacting protein family member 1 | no;no;yes | no;no;no |
| O60343 | TBC1 domain family, member 4 | no;no;no | no;no;yes |
| O60645 | Exocyst complex component 3 | no;no;no | no;no;yes |
| O75937 | DnaJ homolog subfamily C member 8 | no;no;yes | yes;no;yes |
| O95104 | Splicing factor, arginine/serine-rich 15 | no;no;no | no;no;yes |
| O95907* | Monocarboxylate transporter 3 (MCT 3) | no;no;yes | no;no;yes |
| P01876 | Ig alpha-1 chain C region | no;no;yes | no;no;no |
| P04114 | Apolipoprotein B-100 precursor | no;no;no | no;no;yes |
| P06276 | Cholinesterase precursor | no;no;yes | no;no;no |
| P06730 | Eukaryotic translation initiation factor 4E | no;no;no | no;no;yes |
| P08603 | H factor 1 (complement) | no;no;yes | no;no;no |
| P13521 | Secretogranin-2 precursor | yes;no;no | no;no;yes |
| P13866 | Sodium/glucose cotransporter 1 | yes;no;no | no;no;no |
| P13942 | Collagen alpha-2(XI) chain precursor | no;no;no | no;no;yes |
| P15924 | Desmoplakin | no;no;no | no;no;yes |
| P21359 | Neurofibromin | no;no;yes | no;no;no |
| P30622 | CAP-Gly domain-containing linker protein 1 | no;no;no | no;no;yes |
| P46821 | microtubule-associated protein 1B, isoform 1 | no;no;no | no;no;yes |
| P57678 | Gem-associated protein 4 | no;no;no | no;no;yes |
| P59044 | NACHT, LRR and PYD domains-containing protein 6 | no;no;no | no;no;yes |
| P61086 | Ubiquitin-conjugating enzyme E2 K | no;no;no | no;no;yes |
| P83111 | Serine beta-lactamase-like protein LACTB, mitochondrial precursor | no;no;no | no;no;yes |
| Q02224* | Centromere protein E; Centromere autoantigen E | no;no;yes | no;no;no |
| Q03252 | Lamin B2 fragment | no;no;yes | no;no;no |
| Q12834 | Cell division cycle protein 20 homolog | no;no;no | no;no;yes |
| Q13049 | E3 ubiquitin-protein ligase TRIM32 | no;no;no | no;no;yes |
| Q13428 | Treacle protein | no;no;no | no;no;yes |
| Q13838 | HLA-B associated transcript-1 | no;no;no | no;no;yes |
| Q14008 | Cytoskeleton-associated protein 5 | no;no;no | no;no;yes |
| Q14093 | Cylicin-2 | no;no;no | no;no;yes |
| Q14320 | JC5276 HXC-26 protein | no;no;no | no;no;yes |
| Q14534 | squalene monooxygenase | no;no;no | no;no;yes |
| Q14746 | Conserved oligomeric Golgi complex subunit 2 | no;no;no | no;no;yes |
| Q14789 | Golgin subfamily B member 1 | no;no;no | no;no;yes |
| Q14940 | Sodium/hydrogen exchanger 5 | no;no;yes | no;no;no |
| Q14980 | nuclear mitotic apparatus protein 1 | no;yes;no | yes;no;yes |
| Q15149 | Plectin 1 | no;no;no | no;no;yes |
| Q15154 | pericentriolar material 1 [Homo sapiens] | no;no;yes | no;no;no |
| Q15751 | Guanine nucleotide exchange factor p532 | no;no;no | no;no;yes |
| Q15785 | Mitochondrial import receptor subunit TOM34 | no;no;no | no;no;yes |
| Q16771 | Peroxidase (Myeloperoxidase) (Fragment) | no;no;yes | no;no;no |
| Q5JRA6 | Melanoma inhibitory activity protein 3 precursor | no;no;yes | no;no;no |
| Q5T0I2 | Gelsolin | no;no;yes | no;no;no |
| Q86UP2 | Kinectin | no;no;yes | no;no;no |
| Q8WU66 | Thrombospondin-type laminin G domain and EAR repeat-containing protein | no;no;no | no;no;yes |
| Q8WXD2* | Secretogranin-3 precursor | no;no;yes | no;no;no |
| Q92620 | PR16_ Pre-mRNA splicing factor ATP-dependent RNA helicase PRP16 (ATP-depe | no;no;no | no;no;yes |
| Q96SU4 | oxysterol-binding protein-like protein 9 isoform | no;no;no | no;no;yes |
| Q99968 | Translocated Promoter Region | no;no;no | no;no;yes |
| Q9BZ29* | zizimin1 | no;no;yes | no;no;no |
| Q9MY51 | leucocyte antigen A [Homo sapiens] ursor; co | no;no;no | no;no;yes |
| Q9NNX1 | tuftelin 1 | no;no;yes | no;no;no |
| Q9NQC3 | Reticulon 4 | no;no;no | no;no;yes |
| Q9NQX7 | integral membrane protein 3 | no;no;yes | no;no;no |
| Q9NRD1 | F-box only protein 6 | no;no;no | no;no;yes |
| Q9UHB6 | epithelial protein lost in neoplasm beta | no;no;yes | no;yes;yes |
| Q9UQE7 | chondroitin sulfate proteoglycan 6 | no;no;no | no;no;yes |
| Q9Y3R5 | Protein dopey-2 | no;no;no | no;no;yes |
| Q9Y493 | ZAN_ Zonadhesin precursor | no;no;no | no;no;yes |

* Proteins involved in the coagulation/platelet activation and aggregation pathways
