# Supplementary material for: Plasma and Cerebrospinal Proteomes From Children With Cerebral Malaria Differ From Those of Children With Other Encephalopathies
Source: J Infect Dis. 2013 Jul 25;208(9):1494–503. doi: 10.1093/infdis/jit334 (PMC3789566; doi:10.1093/infdis/jit334)
Supplement: Supplementary Data [file supp_jit334_jit334supp_table2.docx]

**SUPPLEMENTARY TABLE 2. *Plasmodium falciparum* proteins identified**

| **Protein family; Protein ID** | **Protein Name** | **CSF_CM_NE_ABM** | **Plasma_CM_NE_ABM** |
| --- | --- | --- | --- |
| ***PFEMP DBL domain; Duffy binding domain*** |  |  |  |
| MAL7P1.187 | erythrocyte membrane protein 1, PfEMP1 (VAR) | no; no; no | no; no; yes |
| MAL7P1.50 | erythrocyte membrane protein 1, PfEMP1 (VAR) | no; no; no | no; no; yes |
| PF07_0051 | erythrocyte membrane protein 1, PfEMP1 (VAR) | no; no; no | no; no; yes |
| PF08_0140 | erythrocyte membrane protein 1, PfEMP1 (VAR) | no; no; no | no; no; yes |
| PF08_0141 | erythrocyte membrane protein 1, PfEMP1 (VAR) | no; no; no | yes; no; no |
| PF10_0001 | erythrocyte membrane protein 1, PfEMP1 (VAR) | no; no; no | yes; no; yes |
| PFB0010w | erythrocyte membrane protein 1, PfEMP1 (VAR) | no; no; no | yes; no; no |
| PFB1055c | erythrocyte membrane protein 1, PfEMP1 (VAR) | no; no; no | yes: yes; yes |
| PFD0995c | erythrocyte membrane protein 1, PfEMP1 (VAR) | no; no; no | yes; no; no |
| PFD1005c | erythrocyte membrane protein 1, PfEMP1 (VAR) | no; no; no | yes; no; yes |
| PFD1235w | erythrocyte membrane protein 1, PfEMP1 (VAR) | no; no; no | no; no; yes |
| PFE1640w | erythrocyte membrane protein 1 (PfEMP1), truncated (VAR1CSA) | no; no; no | yes; no; no |
| PFI0005w | erythrocyte membrane protein 1, PfEMP1 (VAR) | no; no; no | no; no; yes |
| PFL1950w | erythrocyte membrane protein 1, PfEMP1 (VAR) | no; no; no | yes; no; no |
| PFL2665c | erythrocyte membrane protein 1, PfEMP1 (VAR) | no; no; no | yes; no; yes |
| ***Rifin/stevor family*** |  |  |  |
| PF10_0403 | rifin (RIF) | no; no; no | no; no; yes |
| PF14_0002 | rifin (RIF) | no; no; yes | no; no; no |
| PFA0030c | rifin (RIF) | no; no; no | no; no; yes |
| PFI0055c | rifin (RIF) | no; no; no | no; no; yes |
| PFD0050w | rifin (RIF) | no; no; no | no; no; yes |
| PFD0135c | rifin, pseudogene | no; no; no | yes; no; yes |
| PFL0015c | rifin (RIF) | no; no; no | yes; no; yes |
| ***Protein kinase domain*** |  |  |  |
| PF14_0392 | serine/threonine protein kinase, putative | no; no; yes | no; no; yes |
| PFB0150c | protein kinase, putative | no; no; no | yes; no; yes |
| PFB0665w | serine/threonine protein kinase, putative | no; no; no | yes; no; no |
| PFC0060c | serine/threonine protein kinase, FIKK family (FIKK3) | no; no; no | no; yes; no |
| PFC0485w | protein kinase, putative | no; no; no | no; no; yes |
| PFC0755c | cdc2-related protein kinase 4 (CRK4) | no; no; no | yes; no; yes |
| PFF0750w | cdc2-related protein kinase 5 (CRK5) | no; no; no | no; no; yes |
| ***3'-5' exonuclease*** |  |  |  |
| PF14_0473 | exosome complex exonuclease RRP6, putative (RRP6) | no; no; no | yes; no; yes |
| PFA0290w | DNA binding protein, putative | no; no; yes | no; yes; no |
| ***AP2 domain*** |  |  |  |
| PF10_0075 | transcription factor with AP2 domain(s) (ApiAP2) | no; no; no | no; no; yes |
| PF11_0404 | transcription factor with AP2 domain(s) (ApiAP2) | no; no; no | yes; no; yes |
| ***DEAD/DEAH box helicase*** |  |  |  |
| MAL13P1.134 | DEAD box helicase, putative | no; no; no | yes; no; no |
| PF14_0081 | DNA-repair helicase, putative | no; no; yes | yes: yes; yes |
| PFE0205w | ATP-dependent helicase, putative | no; no; no | yes; no; yes |
| PFL1525c | pre-mRNA-splicing factor ATP-dependent RNA helicase PRP2, putative (PRP2) | no; no; no | no; no; yes |
| ***DNA polymerase family A/B*** |  |  |  |
| PF14_0112 | plastid replication-repair enzyme (PREX) | no; no; yes | yes; no; yes |
| PFF1470c | DNA polymerase epsilon, catalytic subunit a, putative | no; no; yes | no; no; yes |
| PF10_0165 | DNA polymerase delta catalytic subunit | no; no; no | yes; no; no |
| ***Heat shock proteins*** |  |  |  |
| MAL7P1.228 | heat shock protein 70, putative (HSP70-x) | no; no; no | yes; no; no |
| PFD0462w | heat shock protein 40 (DnaJ) | no; no; no | no; no; yes |
| PF14_0417 | heat shock protein 90, putative (HSP90) | no; no; no | no; no; yes |
| ***Dynein heavy chain*** |  |  |  |
| PF10_0224 | dynein heavy chain, putative | no; no; no | yes; no; yes |
| PF14_0626 | dynein beta chain, putative | no; no; yes | yes; no; yes |
| PF11_0240 | dynein heavy chain, putative | no; no; no | yes; no; yes |
| ***KH domain*** |  |  |  |
| PF10_0115 | QF122 antigen | no; no; yes | no; no; no |
| PFF1135w | transcription or splicing factor-like protein, putative | no; no; no | yes; no; yes |
| ***RecF/RecN/SMC N terminal domain; SMC proteins Flexible Hinge Domain*** |  |  |  |
| PFD0685c | chromosome associated protein, putative | no; no; no | yes; no; no |
| PFE0450w | chromosome condensation protein, putative | no; no; no | no; no; yes |
| ***RNA polymerase domain*** |  |  |  |
| PFE0465c | RNA polymerase I (RNAPI) | no; no; no | no; no; yes |
| PFC0805w | DNA-directed RNA polymerase II, putative | no; no; no | yes; no; no |
| ***tRNA synthetase class II core domain*** |  |  |  |
| PF11_0270 | threonyl-tRNA synthetase,Threonine--tRNA ligase (ThrRS) | no; no; no | yes; no; no |
| PFL1540c | phenylalanyl-tRNA synthetase alpha chain, putative | no; no; no | no; no; yes |
| PF13_0354 | alanyl-tRNA synthetase,Alanine--tRNA ligase (AlaRS) | no; no; no | yes; no; yes |
| ***Other*** |  |  |  |
| PFA0345w | centrin-1 (CEN1) | no; no; no | yes; no; no |
| PFC0870w | elongation factor 1 (EF-1), putative | no; no; no | yes; no; no |
| PFL1940w | 3-hydroxyisobutyryl-coenzyme A hydrolase, putative | no; no; no | yes; no; yes |
| PF14_0281 | plasmepsin IX | no; no; no | yes; no; no |
| PFE1545c | formin 1, putative | no; no; no | no; no; yes |
| PF14_0334 | NAD(P)H-dependent glutamate synthase, putative | no; no; yes | yes; no; no |
| PFL1155w | GTP cyclohydrolase I (GTP-CH) | no; no; yes | no; no; no |
| MAL8P1.23 | E3 ubiquitin-protein ligase, putative | no; no; no | no; no; yes |
| PFF1140c | ATP dependent DEAD-box helicase, putative | no; no; no | yes; no; no |
| PF07_0035 | Cg1 protein | no; no; yes | no; no; no |
| PFI0700c | met-10 like protein, putative | no; no; no | no; no; yes |
| PF14_0364 | cleavage and polyadenylation specifity factor, putative | yes; no; yes | yes; no; no |
| PFE0270c | DNA repair protein, putative | no; no; yes | no; no; no |
| PFI0960w | dolichyl-diphosphooligosaccharide-protein glycosyltransferase, putative | no; no; no | no; no; yes |
| PFB0350c | serine repeat antigen 3 (SERA3) | no; no; no | no; no; yes |
| MAL13P1.256 | phosphatidylinositol transfer protein, putative | yes; no; yes | yes: yes; yes |
| PFB0130w | octaprenyl pyrophosphate synthase (OPP) | no; no; no | no; no; yes |
| PF07_0071 | queuine tRNA-ribosyltransferase, putative | no; no; yes | no; no; no |
| PF14_0102 | rhoptry-associated protein 1 (RAP1) | no; no; no | yes; no; no |
| PFL1170w | polyadenylate-binding protein, putative (PABP) | no; no; no | no; no; yes |
| PFF1440w | SET domain protein, putative (SET1) | no; no; yes | yes; no; yes |
| PFB0405w | Transmission-blocking target antigen S230 | no; no; no | yes; no; no |
| PFF1265w | oxidoreductase, short-chain dehydrogenase family, putative | no; no; no | yes; no; yes |
| PFF1185w | SNF2 helicase, putative (ISWI) | no; no; no | no; no; yes |
| PFL0785c | signal recognition particle SRP19 (SRP19) | no; no; no | yes; no; no |
| PFC0890w | SNARE protein (SEC22) | no; no; no | yes; no; yes |
| PFF1430c | amino acid transporter, putative | no; no; no | yes; no; no |
| PFD1050w | alpha tubulin 2 | no; no; no | yes; no; no |
| PFE1350c | ubiquitin-conjugating enzyme E2 N, putative (UBC13) | no; no; no | no; no; yes |
| MAL13P1.380 | conserved Plasmodium protein, unknown function | no; no; no | no; no; yes |
| MAL8P1.113 | peptidase family C50, putative | no; no; no | yes: yes; yes |
| PF07_0037 | Cg2 protein | no; no; no | no; no; yes |
| PF14_0530 | ferlin, putative | no; no; no | no; no; yes |
| PFA0280w | asparagine-rich antigen Pfa35-2 | no; no; no | no; no; yes |
| PFB0095c | erythrocyte membrane protein 3 (EMP3) | no; no; no | yes; no; yes |
| PFD0805w | prohibitin-like protein, putative | no; no; no | no; no; yes |
| PFD1045c | erythrocyte membrane-associated antigen | no; no; no | yes; no; no |
| PFL2520w | reticulocyte binding protein homologue 3, pseudogene (RH3) | no; no; no | no; no; yes |
| PFD0740w | cdc2-related protein kinase 3 (CRK3) | no; no; no | yes; no; yes |
| PFD0790c | DNA replication licensing factor, putative | no; no; no | yes; no; no |
| PFL0770w | seryl-tRNA synthetase, putative | no; no; no | yes; no; no |
| PFD0980w | holo-(acyl-carrier protein) synthase, putative | no; no; no | yes: yes; yes |
| PF14_0277 | coatamer beta subunit, putative | no; no; no | no; no; yes |
| MAL13P1.221 | aspartate carbamoyltransferase (atcasE) | no; no; no | yes; no; no |
| MAL13P1.22 | DNA ligase I (LigI) | no; no; no | yes; no; yes |
| PF14_0202 | dynein-associated protein, putative | no; no; no | yes; no; no |
| PFF1450w | sec14-like cytosolic factor or phosphatidylinositol/phosphatidylcholine transfer protein, putative | no; no; no | yes; no; no |
| PFL2375c | cutA, putative | no; no; yes | no; no; yes |
| PFL1110c | cAMP-dependent protein kinase regulatory subunit (PKAr) | no; no; no | no; no; yes |
| PFC0120w | cytoadherence linked asexual protein 3.1 (CLAG3.1) | no; no; no | no; no; yes |
| PFI1580c | DHHC-type zinc finger protein, putative | no; no; yes | no; no; no |
| PF11_0264 | DNA-dependent RNA polymerase | no; no; no | no; no; yes |
